# An analytical framework for breast cancer public policies in Sub-Saharan Africa: results from a comprehensive literature review and an adapted policy Delphi

**DOI:** 10.1186/s12889-024-18937-5

**Published:** 2024-06-07

**Authors:** Gloria Yawavi Gbenonsi, Jessica Martini, Céline Mahieu

**Affiliations:** 1 School of Public Health- Research centre for Social Approaches to Health, Université libre de Bruxelles, Brussels, Belgium; 2 European Social Observatory (OSE), Brussels, Belgium

**Keywords:** Breast cancer, Sub-Saharan Africa, Public policy, Analytical framework

## Abstract

**Background:**

Breast cancer is the leading cause of death from cancer in women and is a major public health problem worldwide. Despite the lower incidence rates of breast cancer in resource-limited settings, especially sub-Saharan Africa, there is a higher mortality rate compared to high-resource countries where the disease has a higher incidence. This makes breast cancer the second deadliest cancer in African women. These poor results reflect the weakness in public health policies. The aim of this paper is to contribute to the effective control of breast cancer by designing a framework for a comprehensive and systemic analysis of these policies in Sub-Saharan Africa.

**Methods:**

This research is based on a literature review that adopted a systematic approach followed by a modified policy Delphi involving breast cancer experts in Sub-Saharan Africa. We included narrative reviews and systematic reviews/meta-analyses published between 2015 and 2022 as well as official documents in the analysis. We integrated the World Health Organization’s health system building blocks with Walt and Gilson’s policy analysis triangle to analyse the information collected and develop our analytical framework.

**Results:**

A total of 22 reviews and documents were included in the study. Sixteen breast cancer experts from Sub-Saharan Africa participated in the first Delphi round, and nine participated in the second round. The different components identified for a comprehensive and systemic analysis of effective breast cancer policies can be classified into policy content divided according to the health system building blocks and related policy processes; individual, organized national and international policy stakeholders; and policy contexts.

**Conclusion:**

This study enabled the design of a framework suitable for the comprehensive and systemic analysis of breast cancer control policies in Sub-Saharan Africa. This framework can be used as a checklist for stakeholders to guide the planning, implementation and evaluation of policies and specific breast cancer control programmes at the national and facility levels.

**Supplementary Information:**

The online version contains supplementary material available at 10.1186/s12889-024-18937-5.

## Introduction

Cancer is one of the leading causes of death worldwide, with 20 million incident cases and approximately 10 million deaths recorded in 2022 [1]. While there are different types of cancers, the most commonly diagnosed cancer in women is breast cancer (11.5%) [2] With 666,103 deaths in 2022, breast cancer is also the deadliest cancer for women [2]. To tackle this public health problem, the World Health Organization launched the Global Breast cancer Initiative (GBCI) in 2021, with the shared goal of reducing breast cancer by 2.5% per year, which over a 20-year period would save 2.5 million lives [3]. This reflects the worldwide commitment to the global fight against breast cancer.

Despite being a global public health problem, low-resource settings register a higher mortality rate (19.2 per 100,000 in 2022) compared to high-resource countries (14.6 per 100,000) [1, 4]. Low- and middle-income countries actually account for more than two-thirds of breast cancer global mortality [1, 5]. This high rate contrasts with the lower incidence these countries record compared to high-income countries, i.e. 38 cases of breast cancer vs. 75.6 per 100,000 women in 2022 [2]. This disparity is even more remarkable in Sub-Saharan Africa (SSA) where the 5-year survival rate is less than 50% vs. 90%.in high-resource countries [1, 4]. Breast cancer in SSA is the first or the second deadliest cancer among women in SSA, after cervical cancer [2].

It should be noted that thanks to advances most types of breast cancer are now curable when detected at an early stage and managed appropriately. Its management, however, constitutes a heavy socioeconomic burden. In addition, the availability and accessibility of the resources needed to adequately manage cancer vary by country and region. A systematic review by Li Sun et al. in 2018 reported that the average financial cost of this care according to the estimations of 15 studies (mostly in high-income countries) can range from $29,724 in stage I to $62,108 in stage IV [6]. Subramanian et al. estimated that in 2018, the cost of breast cancer care in the public sector in Kenya ranged from $1,340 for stage I/II to $1,542 for stage III [7].

These poor survival outcomes in sub-Saharan Africa reflect the fragility of the region’s healthcare systems, which in turn highlights the weaknesses of breast cancer control policies, such as inconsistent interventions, a lack of human, material and financial resources including catastrophic expenditure due to out of pocket payments, a weak political will etc… [10]. These weaknesses lead to considerable delays in both diagnosis and treatment, leaving women with less chance of a cure [8]. Indeed, 77% of women in SSA are diagnosed at the III-IV breast cancer stage, thus further contributing to the low survival rate [9]. A study carried out in 5 sub-Saharan African countries estimated that 28–37% of breast cancer deaths in these countries could be prevented by earlier diagnosis [10]. This situation constitutes a considerable obstacle to increasing life expectancy and to improvising the quality of life. It also presents a considerable limitation to sustainable socioeconomic development, for which the essential role of women has been greatly acknowledged by the United Nations [11].

This study aims to propose a comprehensive and adapted framework for the systemic analysis of breast cancer control policies in SSA. An appropriate analytical framework is indeed important to understand the content of breast cancer policies in this region, how they are developed and implemented, whether they are adapted to the local context and able to strengthen the fight against this disease, and what actors are involved. To the best of our knowledge, no comprehensive and systemic analytical tool for breast cancer policies has been developed yet at the global level, particularly one adapted to SSA.

## Methods

### Literature review and development of a preliminary framework

This study was based on a literature review that adopted the systematized approach promoted by Saracci et al. and the National Institute of Public Health of Quebec [12, 13]. This approach aims to reduce biases related to study selection and quality and ensure the transparency and reproducibility of the research strategy.

### Data sources and research strategy

We included both scientific and grey literature aiming to provide comprehensive recommendations for effective breast cancer control policies in SSA. The Medline (PubMed), Cochrane review, and Scopus databases were our three main data sources for identifying relevant literature. In addition, we searched for official documents from the World Health Organization (WHO), the International Agency for Research on Cancer (IARC), the Breast Health Global Initiative (BHGI) and the African Organization for Research and Education on Cancer (AOREC). The following keywords were combined to obtain several search equations according to the databases: “Breast cancer”; “Breast carcinoma”; “Breast neoplasm”; “Breast Tumour”; “Health care policy”; “Breast cancer policy”; “Breast cancer guideline”; “Sub-Saharan Africa’’; “Low- and middle-income countries” ; “Service delivery” ; “Health workforce”; “Health information system”; “Medicines”; “Technologies”; “Financing”; “Leadership”; “Governance”. Details and the equations developed are provided in additional file 1.

### Eligibility criteria for literature

We included literature according to the following eligibility criteria: literature/systematic review and meta-analysis published between 2015 and June 2022; official reports published by the WHO or IARC, BHGI, AOREC; geographical focus on the entire SSA or one of its subregions with at least two countries and/or low- and middle-income countries in general but including SSA countries in the results; content addressing different personal/systemic factors that influence breast cancer morbidity and mortality in SSA and highlighting elements or recommendations for effective breast cancer control in SSA.

### Study selection and data extraction

The studies identified through the search were transferred to Zotero software, which enabled us to organize the references and address duplication. Eligible papers were identified using the Preferred Reporting Items for Systematic Reviews and Meta-Analyses (PRISMA) flowchart [30], which was adapted for our review. After the selection, an extraction form developed in Microsoft Excel was used to collect the relevant information identified in each document.

### Quality assessment of included reviews

The quality of the included systematic reviews/meta-analyses was assessed using the Joanna Briggs Institute (JBI) critical appraisal checklist for systematic reviews [14] (see Additional file 2). Narrative literature reviews were evaluated with the Scale for the Assessment of Narrative Review Articles (SANRA) [13, 15] (see Additional file 3).

### Data analysis

**Fig. 1:**
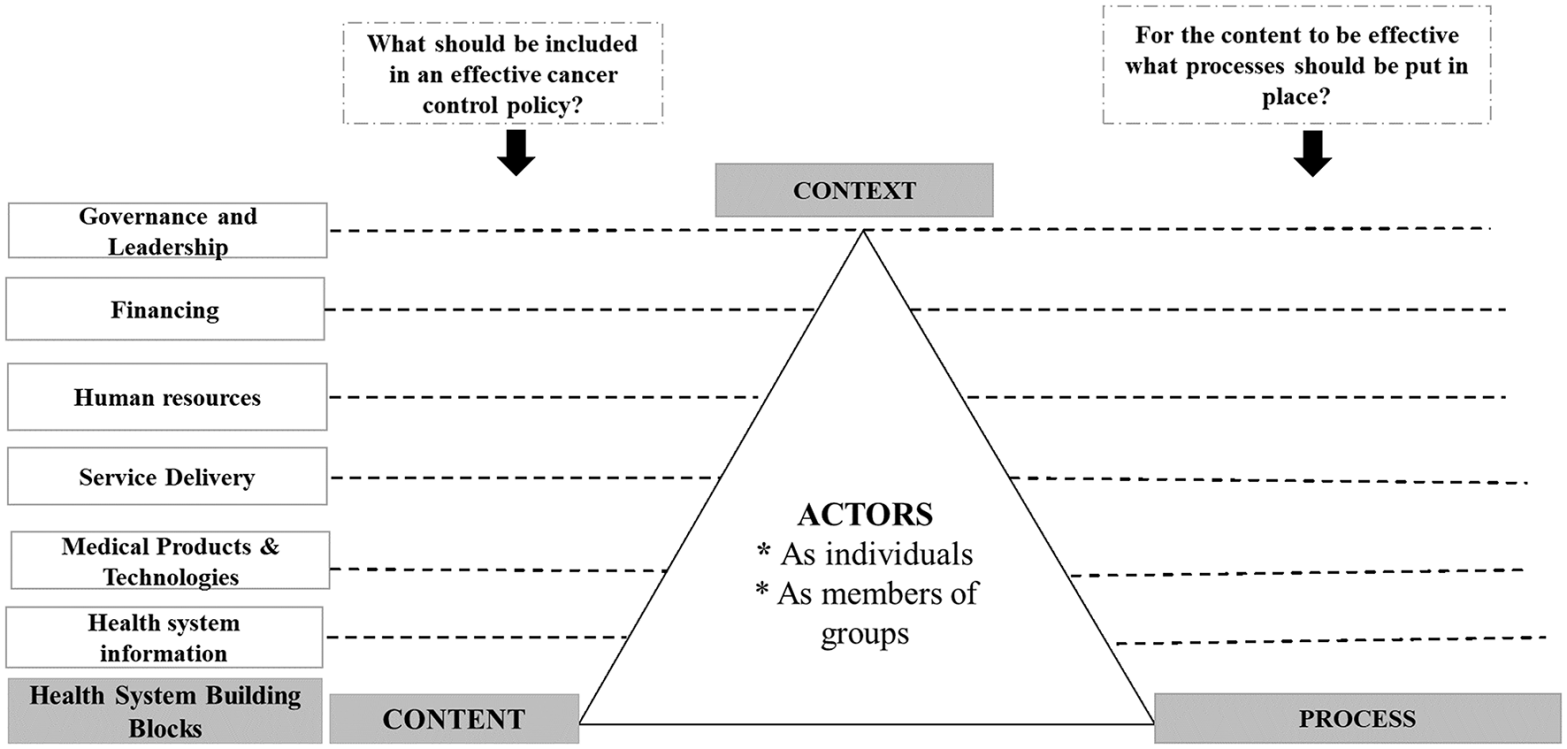
Combination of health system building blocks of WHO [17, 18] and The Policy Analysis Triangle of Walt and Gilson [16]

The two main frames of reference used to analyse the extracted data and realize our analytical framework were the Walt and Gilson analysis triangle [16] and the WHO’s health system building blocks [17, 18]. These two frameworks were combined to identify all relevant components for an effective breast cancer control policy (Fig. 1). These components were grouped as policy content, policy process, stakeholders, and policy context. According to Walt and Gilson, the content of a health policy refers to all elements that improve the health system organization and increase the access to, coverage of, and use of health services. The WHO invites countries to strengthen their health systems by considering their building blocks, namely, governance and leadership, financing, human resources, service delivery, medicines and technologies, information systems and population. Therefore, the different items found in relation to policy content in the included papers were categorized according to each of these building blocks. Likewise, we grouped all items found in relation to processes according to the different building blocks. We classified as policy content all items that could answer the question, “What should be included in an effective cancer control policy?‘’ The policy “processes” answered the following question: “How can the content of the policy be implemented?” It should be noted that content and process are often linked, and that this distinction is most often proposed for analytical purposes. This is also based on our own interpretation since most of the included papers did not directly distinguish them in these terms. Contexts are the different external conditions that can shape the policy and that should be considered for implementation. Finally, individual or collective stakeholders were classified into national stakeholders (any person or organization at the national level involved in or concerned with breast cancer policy) and international stakeholders. As the population is part of the building blocks of a health system, not only as a beneficiary but also as a full stakeholder at the centre according to the WHO [18], we included it in the ‘’stakeholders’’ component, which is also central in Walt and Gilson’s triangle [16].

Our framework has the following hierarchic structure: (1) four main components refers to the content, the process, the stakeholders and the context of the policy; (2) specific items were identified under each component (the items of content and process were classified according to the health system building blocks); and (3) subitems were identified under each item.

### Participant observation and adapted policy Delphi: review of the literature-based framework

In October 2022, the first author (GYG) attended the World Cancer Congress in Geneva organized by the International Union Against Cancer. During the congress, GYG participated in workshops on breast cancer in Africa where many items found in the literature-based framework were discussed with key actors.

The literature-based framework was then submitted to experts with relevant experience in the fight against breast cancer in SSA through a two-round adapted policy Delphi [19]. Policy Delphi is a variant of the traditional Delphi technique but shares principles such as the anonymity of experts and multi-round validation. Its main objective is not to seek consensus but to bring out different points of view from a group of experts on a policy issue [19, 20]. In other words, our objective by using this approach was, on the one hand, to ensure that all the elements required for an effective breast cancer policy in SSA are identified. On the other hand, to ensure that experts provided feedback on each item of the proposed analytical framework, specially to know if they had additional items to add or remove from what was proposed, and their comments on the presentation of the framework in general. This helped to readapt the framework to SSA’s realities and context.

These experts were identified among the participants in the World Cancer Congress, key authors in the literature and the snowball method. In all, 25 experts with experience in the West, Central, East and South African regions were invited to participate in this survey, including clinical oncologists, researchers, cancer programme managers, governmental and nongovernmental organization managers, and breast cancer advocates or survivors living in and/or working on SSA. In the first round, experts were invited to complete a LimeSurvey questionnaire from December 2022 to January 2023. In the second round, a summary report including feedback from the first round was sent to the experts in Word form for review and approval between March and April 2023. In general, there was no dissensus between the experts’ feedback. Additional file 4 lists the experts who agreed to have their names disclosed in this research.

## Results

### Characteristics of the included literature

**Fig. 2:**
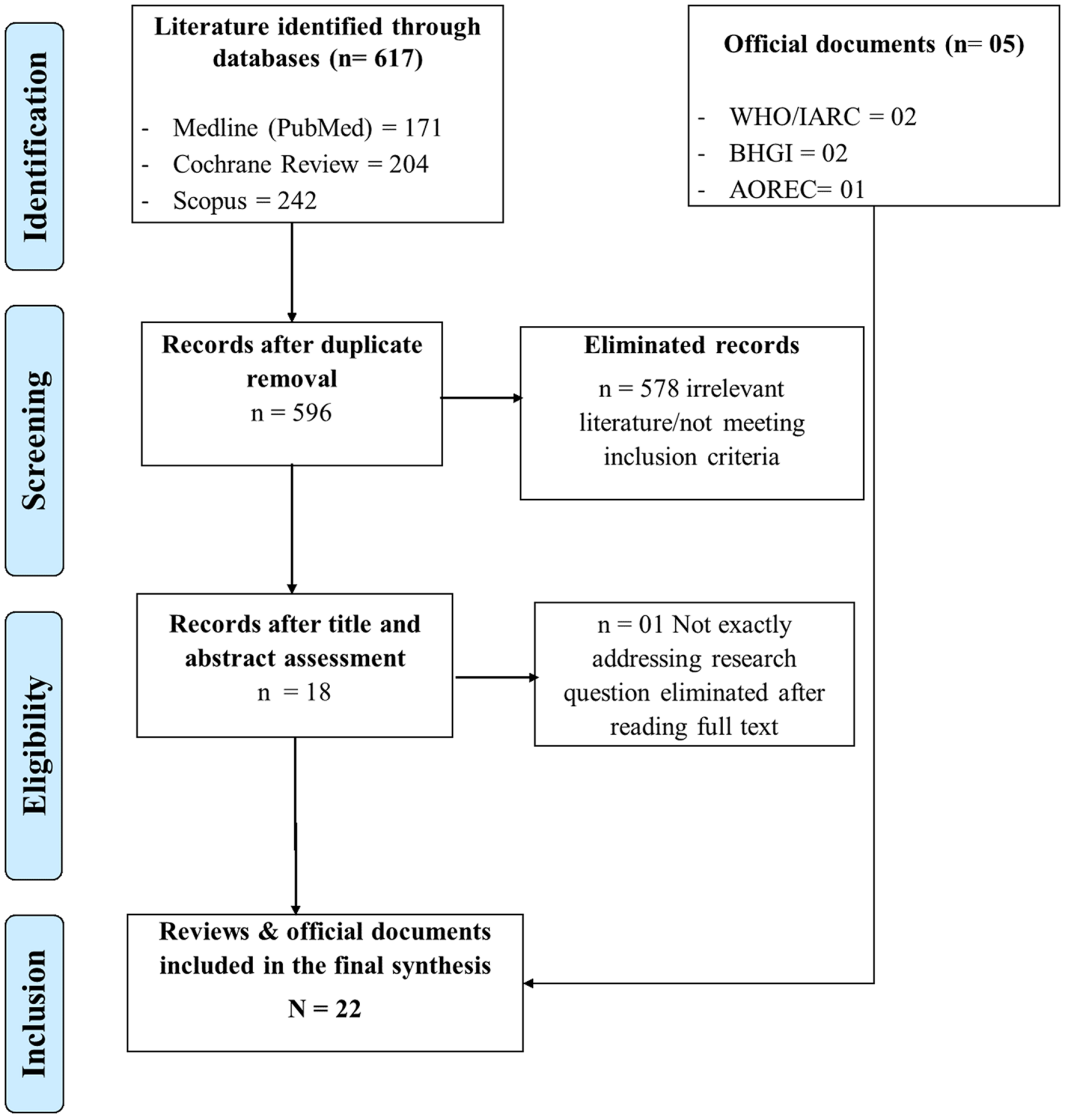
Modified Prisma flow diagram for articles and documents selection

A total of 617 studies were identified through the preselected databases, of which 17 reviews were finally retained, including six systematic reviews/meta-analyses, one scoping review, and ten narrative reviews. In addition, we identified and included five official documents from the WHO, IARC, BHGI and AOREC [21–25] (see Fig. 2).

### Characteristics of Delphi participants

Sixteen experts participated in the survey in the first round, three of whom did not complete the full questionnaire. Nine participants took part in the second round, with two new participants who had not participated in the first round. One of these two participants did not complete the sociodemographic data. More than half of the participants had more than 6 years of experience with breast cancer in SSA. Table 1 presents the details of the profile of the participants.

**Table 1:** Delphi participants socio-demographic characteristics(*n* = 14)*

Characteristics		Number
**Region of expertise**	**Country**	
West Africa	Ghana, Guinea, Nigeria, Benin, Ivory Coast, Burkina Faso, Sierra Leone	7
Southern Africa	Botswana, Zambia	2
Central Africa	DR-Congo, Congo-Brazzaville,	2
Whole of Africa	-----	3
Gender		
	Female	8
	Male	6
Age		
	24–34	1
	35–44	4
	45–54	8
	≥ 55	1
Years’experience with cancer		
	> 10	5
	6–10	4
	1–5	5
Areas of expertise		
	Medical oncologists	7
	Scientific researchers	7
	Breast cancer survivors & Advocates	2
	Director of Cancer Control Organization including NGOs	4
	Cancer program designers /evaluators Coordinator/manager	5

*The socio-demographic data of 4 participants are not known, as they did not complete the entire questionnaire

### Components of an effective breast cancer control policy in Sub-saharan Africa

**Fig. 3:**
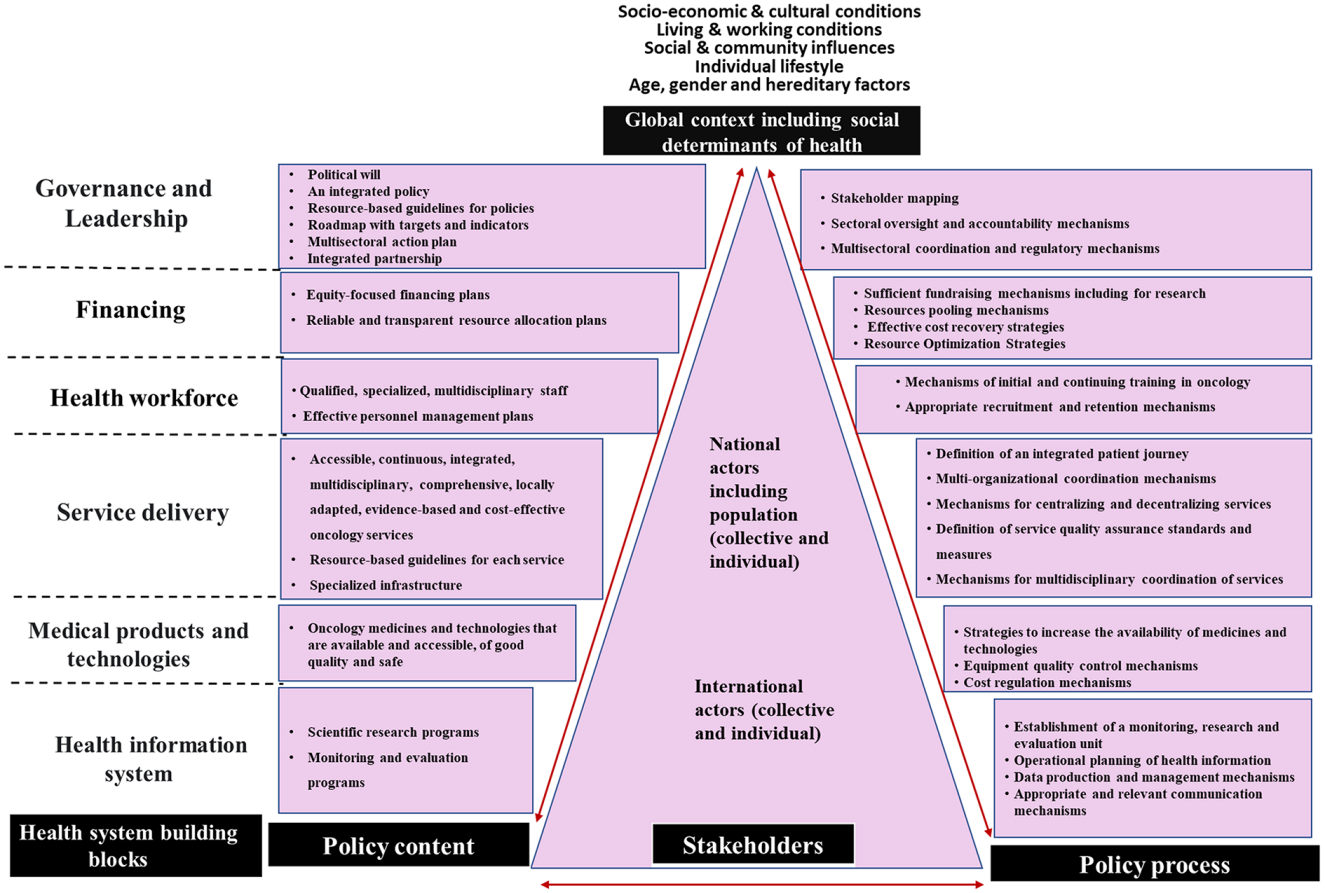
Proposed analytical framework for breast cancer policies in Sub-Saharan Africa

Figure 3 presents the analytical framework resulting from the literature review and the policy Delphi survey. It summarizes the four main components of an effective breast cancer public policy, namely, stakeholders, policy content, policy process and context. The items identified for policy content and process were organized according to the health system building blocks (governance and leadership, financing, health workforce, service delivery, medical products and technologies, and health information system). Further details are provided in the following sections.

### Stakeholders that should be involved

Our results show that different stakeholders should be considered in the planning and implementation of breast cancer policies in SSA. They can be classified into two main groups. The first group includes national stakeholders, most often organized collectively through public organizations (e.g., ministries, state agencies) or private organizations (e.g., various civil society associations, patient advocacy groups, health professional organizations, media) [22, 24–26]. The second group includes international stakeholders organized collectively as public or private technical and financial partners (e.g., international governmental and nongovernmental organizations) [22, 23, 26–28]. It should be noted that for both groups, stakeholders can also act on their own as individuals, and their individual characteristics may influence the way policy is developed and implemented [18, 19].

All these stakeholders play an important role in the development and implementation of breast cancer policy, with a steering role for the Ministry of Health and other relevant ministries [23]. In particular, the Delphi participants emphasized that the government of each country is the primary actor responsible for ensuring sufficient resources for cancer control with support from international and local partners. Financial partners, particularly international partners, are particularly important to increase financing for breast cancer control in SSA [22, 26, 28].

Both the Delphi participants and the literature recommend involving a range of national stakeholders, including community leaders, traditional healers, and patients’ caregivers. In particular, involving beneficiaries allows a beneficiary-centred approach and is expected to enhance their adherence to programmes [24, 29]. For BHGI, change can often involve programme champions such as cancer survivors, family members, community religious leaders, and celebrities leading change [22]. Advocacy groups organized by these champions can contribute to advocacy efforts; these groups are active in Uganda, for instance [22, 26]. In terms of scientific experts, international and national actors, such as medical and surgical oncology societies and universities, should be involved.

These stakeholders could be involved at different stages of the policy process and in different areas depending on their expertise and the issues at stake. The Delphi participants recommended involving them gradually in policy planning and implementation according to their respective roles and expertise. This can be decided on a case-by-case basis mainly depending on the purpose to be achieved.

### Policy content and processes required for breast cancer control in SSA

#### Governance and leadership building blocks

In terms of policy content, four main items emerged from our study in relation to the governance and leadership of a breast cancer policy: political will, an integrated approach to cancer prevention and control, clear roadmaps and multisectoral interventions.

Concerning “political will”, the BHGI and Yip et al. affirm that targeted and sustained government support is crucial to provide, subsidize and regulate cancer care services [22, 27]. The need for political will was also emphasized by the Delphi participants.

“An integrated approach” to cancer prevention and control was explicitly cited by the resolution on cancer adopted at the 70th General Health Assembly in 2017 in Geneva [23, 25]. This approach consists of including breast cancer programmes in existing health system platforms and thus benefits from pooled resources (human, material, financial, etc.). Zambia and Tanzania are cases in point since breast cancer programmes were built into a cervical cancer control programme [28]. In addition, the integrated approach involves the prioritization of breast cancer in the national health plan while adopting “resource-based guidelines” for national-level strategies such as those developed by the BHGI. These guidelines are stratified according to the different levels of available resources [23, 30].

A third key item is the elaboration of a “roadmap with objectives and indicators” (including specific, measurable, achievable and time-bound objectives) to guide strategy operationalization and to enable effective communication with stakeholders [23, 25, 30–32].

Finally, breast cancer control requires “multisectoral action”, i.e., planning and implementation that extends beyond the national health system and involves multiple stakeholders such as ministry of education, agriculture, culture, financing etc [22, 23, 25, 27, 29, 30]. In this respect, the Delphi participants suggested going beyond simple stakeholder consultation and establishing a concrete partnership plan that includes public‒private partnerships and clearly defines the role of each actor in the policy.

In terms of the policy process, three main items emerged from our results: a prior mapping of all stakeholders, sectoral oversight, and accountability mechanisms, as well as multisectoral coordination and regulation mechanisms.

A prior “mapping of the stakeholders” concerned by the policy is crucial to ensure their effective engagement and to identify the role that each of them could play as well as conflicts of interest [23, 30].

Concerning the “sectoral oversight and accountability mechanisms”, these are essential to monitor and evaluate whether interventions are implemented properly and whether real resources are deployed. This could refer to the creation of National Cancer Control Councils [22], the establishment of a unit or department within the Ministry of Health for noncommunicable diseases, including breast cancer [25], and on-site visits and the use of monitoring tools such as implementation checklists proposed by the BHGI (checklists of resources needed to provide safe and effective treatment) and WHO (priority medical devices for cancer management) [23, 30].

In addition, “multisectoral coordination and regulation mechanisms” could help to ensure policy coherence and the supervision of all involved actors as well as those engaged beyond the health sector [22, 23]. These mechanisms can refer to regular meetings. Both the WHO and the BHGI advocate the creation of a national multisectoral commission or agency to oversee commitment to breast cancer control, policy coherence and the accountability of sectors beyond health [25, 25].

#### Financing building blocks

Effective financing is about making available the necessary funds for breast cancer control. In terms of content, this requires two main items: financing plans and reliable and transparent resource allocation plans.

“Financing plans” are needed to ensure equity and protect patients from catastrophic spending [22, 24, 27, 32–37]. Horton et al. report that single-payer funding is a good strategy to ensure equity. Public system involvement could increase the obligation of both public and private service providers to comply with uniform standards of coverage and care [28].

In addition, Mutebi et al. suggest that “reliable and transparent resource allocation plans” increase the efficiency and coverage of real needs [30]. In this sense, guidelines for the diagnosis and treatment of breast cancer and an evidence-based investment portfolio are essential to guide resource allocation and save budgets [22, 35].

In terms of process, the following items were found: sufficient fundraising mechanisms, effective cost recovery strategies, optimization of existing resource strategies and resource pooling mechanisms.

“Sufficient fundraising mechanisms” are needed to provide the required funds for breast cancer care. These involve mobilizing partners, searching for private and external support, and advocating for investment, both with governments and with international organizations and nongovernmental organizations [22, 28]. The Delphi participants suggested the introduction of taxes on products (e.g., tobacco, sweet and salty drinks).

Similarly, “effective cost recovery strategies” are needed to ensure that services are affordable for all patients. On the one hand, this may refer to the adoption of a progressive cost-recovery model adapted to patients’ needs and to increasing government subsidies [22, 28, 37], such as in Zambia, where the government covers breast cancer screening and treatment costs in public facilities [28]. For the Delphi participants, it is important to adopt laws for free gynaecological cancer care and allocate a budget to the Health Ministry for intervention implementation. On the other hand, cost recovery could be achieved through the development of national, private or community-based social insurance and the integration of oncology services into universal health coverage [22, 24, 28, 32, 33, 35]. Ghana, for example, introduced a national health insurance scheme in 2003 that covers breast cancer [28]. The need for universal health coverage was particularly emphasized by the Delphi participants.

In addition, “strategies to optimize existing resources” were identified as required for breast cancer control. These include integrating breast cancer policy into existing health platforms so that new interventions can benefit from resources already deployed for other interventions [25, 28, 32].

Finally, the need for “resource pooling mechanisms” was suggested by the Delphi participants as key to providing public policy with a common package of resources. This is important to ensure effectiveness and avoid system fragmentation because SSA countries may have several and separate financing sources.

#### Human resources building blocks

In terms of policy content, the following items are required for effective human resources: specialized and qualified staff with multidisciplinary expertise and effective human resources management plans.

Effective breast cancer services need “specialized and qualified staff with multidisciplinary expertise” because multidisciplinary teams improve both patient satisfaction and clinical outcomes. These include specialist medical staff such as oncologists, surgeons, pathologists, radiotherapists, radiologists, and imaging technicians as well as medical and paramedical staff qualified in oncology care, including supportive care, such as general practitioners, psychologists, nutritionists, nurses, and midwives [21–24, 27, 30, 32, 34–36]. In addition, the Delphi participants highlighted the importance of “patient navigators” who facilitate the patient’s journey, further enhance the integrated patient pathway established and reduce loss to follow-up.

Furthermore, “effective human resources management plans” are essential to ensure the availability and geographical accessibility of breast cancer staff [22]. In this respect, the Delphi participants stressed the need to ensure the availability and accessibility of qualified, multidisciplinary staff.

Concerning the policy process, the following components emerged in relation to human resources: mechanisms for initial and continuing training and mechanisms for staff recruitment and retention.

“Initial and continuing training mechanisms” targeted towards breast cancer and adapted to local contexts are crucial to achieve access to qualified and available personnel [22, 26, 28, 30, 32–34]. Training involves staff from primary to tertiary care structures and could be provided locally or abroad to allow experience exchange [22]. National medical and paramedical training programmes should provide basic training in molecular oncology, including clinical breast examination techniques, as well as training in oncology specialties and palliative services [22, 23, 30, 36]. Continuing training for working staff could address, for example, the importance and methods of early detection and diagnosis; performing biopsy and processing samples; chemotherapy administration; fundamentals of multidisciplinary care; and performing a standard mastectomy and full axillary node dissection for general surgeons to ensure access to adequate surgical care while waiting for a sufficient number of surgical oncologists [22, 23, 28, 30, 32, 33, 38, 39]. For the Delphi participants, continuing training could be accomplished through mentoring, e-learning, and consensus meetings between practitioners. Concerning training abroad, the BHGI suggests that it should preferably be conducted through exchange programmes between countries with similar resources to ensure resource equivalence, but this does not preclude exchanges with countries with higher resources [22], such as the Ghanaian pathologists trained in Norway as part of the Ghana-Norway collaboration aimed at re-establishing surgical pathology at the Komfo Anokye Teaching Hospital in Ghana [22].

In addition to training, appropriate “recruitment and retention mechanisms” are essential. This includes, for instance, integrating primary health care workers and community-based workers in breast cancer awareness, screening, and diagnosis [22, 24, 25, 30, 38] and recruiting and training volunteers to increase accessibility to awareness and early detection services, especially in remote areas, as successfully adopted in countries such as South Africa, Tanzania and Sudan [21, 26, 36]. Mechanisms to motivate and retain local staff and to attract foreign-trained staff include financial incentives, salary payments or the provision of adequate equipment for their required work [22]. The Delphi participants also stressed the importance of health worker recruitment and retention mechanisms in SSA.

#### Service delivery building blocks

In terms of policy content, our findings related to service delivery highlight the need for oncology services that are accessible, continuous, integrated, multidisciplinary, comprehensive, community-oriented, evidence-based, and cost-effective, resource-based guidelines that regulate the different services, and specialized infrastructures.

Comprehensive oncology services include promotion and primary prevention, early detection, diagnosis, treatment, and palliative care [21–23, 25, 26, 30, 32, 35, 36, 40]. In a progressive implementation approach due to limited resources, the Delphi participants tended to prioritize **“**promotion and primary prevention services**”**. Promotion and primary prevention are essential to inform the population and induce women to adopt appropriate attitudes that contribute to reducing the incidence of breast cancer. The term **“**promotion**”** refers to protective factors against breast cancer: e.g. promoting physical activity and a healthy diet. “Primary prevention” is all actions aimed at reducing the incidence of breast cancer, and therefore focuses on reducing risk factors such as smoking and alcohol [41, 42].

Early detection strategies in SSA should involve community awareness and screening programmes [21–26, 29, 30, 33, 43]. Concerning breast screening, different breast screening strategies are feasible, including breast self-examination (BSE), clinical breast examination (CBE) by a practitioner, and mammography. There is also, the clinical downstaging which is an alternative to these screening strategies [21, 22, 24, 29, 33, 36, 43]. Indeed, clinical downstaging is intended for symptomatic women and combines CBE and public awareness. It is referring to the process of ensuring that symptomatic women (with a palpable cancer or other clinically detectable symptom), are diagnosed at earlier stage. It is distinct from screening tests which target asymptomatic women (CBE, BSE, mammography, ultrasound…). It is allowing the disease to be detected at a less advanced stage in the absence of screening [39].

Nevertheless, because most of these strategies, especially mammography, have been predominantly evaluated in high-resource countries, it is crucial to avoid simply prescribing what works in these contexts without considering its merits and likely effectiveness in the SSA context. This calls for evidence-based choices in each context [25, 40, 44]. For the BHGI, breast self-examination, clinical breast examination and clinical downstaging in addition to public awareness programmes could be alternative approaches to mammography screening in SSA [22]. For example, clinical breast examination has been successfully adopted in Malawi and Ghana [35, 37, 39], and clinical downstaging has been demonstrated in Tanzania, Sudan and Malaysia [39]. Even though CBE and BSE are low-tech approaches, they are important in helping to reduce the diagnostic delays faced by SSA countries. Indeed, the need for contact with a professional to carry out the clinical examination enables women with clinical abnormalities to be put in touch with healthcare at the time the abnormality is detected. Concerning BSE, Eleanor Black & Robyn Richmond concluded in their review that, although current evidence does not support it as an approach to breast cancer screening, teaching it at an individual level could improve awareness and lead to earlier diagnosis in settings where most women present with an advanced stage [39].

The evidence suggests that early diagnostic services that combine the “triple test” of clinical breast examination, diagnostic imaging and tissue pathology should be part of service delivery in SSA [23]. The BHGI particularly reported that the “triple test” has proven reliable with high sensitivity and specificity, even in the context of resource-limited countries such as SSA [23]. Regarding diagnostic imaging, Kiven Erique Lukong et al. reported that echography is cost-effective and used in SSA countries such as Nigeria and Uganda [37]. Finally, confirmation of breast cancer diagnosis requires adequate anatomopathology laboratories that allow for quality processing of the sampled tissues [21–23, 25, 27, 30, 35, 36]. In addition to diagnosis confirmation, laboratories must be able to perform staging and subtype evaluation of the cancer, which are the pillars of the treatment of this disease [22, 23, 30, 40, 45].

Stages I-III of the disease require curative treatment, including breast surgery, radiotherapy and systemic treatment [22, 23, 27, 30, 35–37, 40]. Surgical treatment of breast cancer requires the provision of adequate support services, including operating rooms, anaesthesia, and technical assistance [23, 30]. Breast radiotherapy plays an essential role in both curative and palliative treatment [30]. Systemic therapy requires appropriate guidelines for drug selection and administration to ensure that all patients receive the right drugs at the right doses [22]. Palliative care and pain management services, including support and survivorship plan, are a priority for patients with advanced disease (stage IV) but are also essential for all diagnosed patients. These services use pharmacological and nonpharmacological approaches, including psychosocial and spiritual support, to provide comprehensive patient-centred care [22, 23, 25–27, 30]. In addition, the Delphi participants suggested the integration of transversal services, such as pastoral services and patient support groups, for the management of patients.

Resource-based guidelines are essential to regulate different oncology services [23, 27, 29, 30, 36]. For example, the BHGI guidelines provide a stratified, resource-sensitive framework for overcoming barriers to implementing breast health interventions when resources are limited [46].

Finally, breast cancer services require “adequate and specialized infrastructures”, especially anatomopathology laboratories and systemic therapy structures (23,24,31).

Different policy processes were found in association with oncology service implementation, including an integrated patient pathway; mechanisms for multiorganizational coordination, services centralization and decentralization, and multidisciplinary coordination; and quality assurance standards and measures.

Concerning the “integrated patient pathway”, it is essential to provide appropriate patient orientation that easily enables navigation through the health care system. This refers to a definition of a clear pathway with targets for the desirable time interval between episodes of management [23–25, 30, 32, 34, 36]. In this respect, the BHGI has developed a universal patient pathway that could inspire countries worldwide, including in SSA [23]. In a progressive implementation approach due to limited resources, the Delphi experts prioritized the “integrated patient pathway”.

“Multiorganizational” collaboration requiring effective “coordination mechanisms” is essential to make the patient pathway truly operational while facilitating dialogue between providers. This calls for a definition of the role of each primary-, secondary- and tertiary-level centre in the continuum of care; the creation of health care networks in which centres of excellence are linked to each other and to peripheral centres in rural and surrounding areas; good communication facilities (e.g., mobile phone connections, electronic teleconferencing, video conferencing), and the implementation of task-shifting models such as those used by Rwanda and Kenya [23, 30, 35].

“Centralization and decentralization mechanisms” are required to improve the accessibility and quality of oncology services [22, 23, 29]. Centralization is necessary to address the fragmentation of the system, the services, and staff shortages. It can be achieved by creating accredited centres of excellence that pool together all essential services, serve as research and training centres, and establish a standard of care that is nationally recognized [22, 23, 30]. On the other hand, decentralization mechanisms are also necessary to address the potential issue of geographical inaccessibility resulting from centralization [22, 23, 30]. Decentralization can be achieved by integrating primary health care facilities for oncology services and establishing public‒private partnerships [22, 25, 28, 30, 34]. Kenya, for example, has adopted partnerships to decentralize their comprehensive cancer care centres [30]. Similarly, South Africa has illustrated this through partnerships with local transport companies to improve access to care [30].

The adoption of “quality assurance standards and measures” is necessary to ensure the consistent application of guidelines and care protocols throughout a patient’s journey, especially at decentralized centres [30, 36]. Performance and quality measures have been developed in Tanzania, South Africa, and Rwanda and could be adapted to each SSA country [30, 47]. Ensuring quality care requires periodic or continuous monitoring, including regular quality checks that assess, for instance, practitioners’ knowledge of the patient journey, proficiency in screening and diagnosis techniques, and the ability to identify patients who require additional diagnostic services, the adequate implementation of care guidelines and protocols, and conformity of pathology reports, which should be precise and follow regulatory body guidelines such as the College of American Pathologists [22, 23, 27, 30, 36]. In addition, national accreditation and certification programmes could help maintain the integrity and quality of care at the centre level [27, 30]. Checklists can ensure the availability of necessary resources for safe and effective therapy before patients are managed [30]. Collaboration with the Quality Assurance Team for Radiation Oncology (QUATRO) is recommended for radiation therapy services in SSA countries [22].

“Multidisciplinary coordination mechanisms” are essential at the health care system level. These mechanisms could include the creation of multidisciplinary committees and regular meetings with all breast cancer staff [23, 30]. Multidisciplinary meetings have many advantages, such as providing a forum for exchanging and properly following treatment guidelines at the institutional, national, or international level. These meetings also provide participants with up-to-date information on new breast cancer treatment strategies [23, 30].

#### Medicine and technology building block

In terms of policy content for effective breast cancer control policy in SSA, the main item found in relation to medicines and technologies is the “availability of equitable access to safe, quality essential medicines and technologies”. This requires adequate equipment and technology for breast cancer screening, diagnosis, and management [22, 23, 27, 28, 30, 32, 35, 37, 39]. Essential oncology drugs, including painkillers, should be made available and easily accessible to all patients in need [22, 23, 25]. For this purpose, the WHO provides countries with a list of essential and specific drugs for curative cancer treatment and pain management [23, 28, 30].

Concerning the policy process, three items were found: mechanisms for medicines and technologies availability, mechanisms for quality control, and mechanisms for cost regulation.

The “mechanisms to increase the availability” of essential medicines and technologies for breast cancer control include the manufacture of simplified radiotherapy equipment that is adapted to available resources in SSA countries [22] and the use of checklists such as WHO lists of essential drugs and technologies in oncology [23, 28, 30].

“Quality control mechanisms” are important for equipment used in breast cancer control. According to the literature [22, 27], when budgets are limited, funding should be directed towards maintaining existing equipment instead of purchasing new, expensive machines. Maintenance contracts and collaboration with international organizations such as QUATRO can ensure regular maintenance and calibration of radiation equipment to maintain the quality of treatment offered to patients [22, 27].

In addition, “mechanisms for regulating drug costs” are needed to ensure affordable treatment for all patients regardless of socioeconomic status, as suggested by the BHGI. These mechanisms involve allocating a balanced proportion of national cancer control budgets to the purchase of drugs [22]. Moreover, Yip et al. report that cost regulation could be achieved through partnerships with pharmaceutical companies [27]. The Delphi participants also proposed the introduction of legislation for a public‒private partnership for cost regulation.

#### Health information system building blocks

A performing health information system is essential because all interventions should be evidence-based from planning to implementation [22, 23, 27, 32, 35]. In this respect, three main items of policy content emerged from our findings: research programmes, monitoring and evaluation.

Scientific research programmes that adopt quantitative, qualitative, or mixed methods are essential for targeted and resource-based breast cancer control strategies [22, 25, 35]. The literature flags several areas of research, including the assessment of the epidemiological and socioeconomic burden of the disease [22, 35], situational analysis of the population’s knowledge, attitudes, and practices related to the disease [27], biological and histological characteristics of breast cancer cells in different population groups [44, 45], and the mapping of available resources [22, 23, 25].

Monitoring and evaluation programmes are crucial for tracking the implementation of interventions, making necessary adjustments, monitoring changes in indicators, and evaluating the results achieved. These programmes involve continuous or episodic monitoring of morbidity and mortality indicators and breast cancer risk factors, and increased surveillance of high-risk populations [25, 27]. Evaluation should also include system performance, cost-effectiveness of interventions, assessment of implementation costs and the assessment of stage-specific survival [22, 27, 30].

In terms of the policy process, a breast cancer control policy requires the following items: a unit with a responsible person, operational health information planning; mechanisms for data production and management, and mechanisms for appropriate communication and funding.

To allow the information system to perform its functions, it is essential to establish a “unit with a responsible person” for the monitoring, research and evaluation of breast cancer strategies in each SSA country [25].

The implementation of research, monitoring, and evaluation programmes requires “operational health information planning”. For this purpose, the BHGI proposes using appropriate measures and frameworks to guide interventions, such as the WHO health system building blocks [23].

The “data production mechanisms” required for research, monitoring, and evaluation could include periodic surveys and routine systems for collecting information on breast cancer morbidity and mortality [22, 25]. Standardizing information collection tools through the development of hospital or population-based cancer registries is essential [22, 23, 25, 27, 35, 36, 39, 44], and predefinition of the data to be collected is necessary to ensure completeness. Mandatory data to be collected should include information on the stage and size of the tumour at diagnosis and data for assessing breast cancer morbidity and mortality. Population-based cancer registries have been established in some parts of SSA, such as Gambia, Uganda, and Zimbabwe [23, 45]. In addition, the Delphi participants suggested “data management mechanisms” as an essential item of the health information system.

Regarding health information dissemination to different stakeholders, the BHGI emphasizes the need for “appropriate and relevant communication mechanisms” [23]. Indeed, clear and accurate communication tools facilitate dialogue and guide evidence-based policy-making. These could include knowledge summaries to translate evidence into practice and microsimulation models to estimate the impacts of selected interventions [23].

Finally, “funding mechanisms” for scientific research are essential to ensure effective policy implementation [22].

### Contextual component to be considered

To ensure policy success, it is crucial to consider the specificities of the local contexts in which they will be implemented. Failure to anticipate contextual factors could compromise beneficiaries’ adherence to programmes as well as stakeholders’ commitment, and well-designed protocols may be damaged [22, 36, 39]. The Delphi participants stressed the importance of understanding the context broadly to include all social determinants of health as well as the health system context and capacity.

The literature points out different types of contexts that need to be considered, especially sociocultural, religious, demographic, and economic contexts. Social and cultural context refers to the educational level of the population, including the beneficiaries [24, 29, 33], their perceptions and knowledge about breast cancer [22, 27, 29, 33], geographical areas of intervention (rural or urban) [29, 36], and the marital status of women [29]. The religious context includes different beliefs [22, 27, 29, 33, 35, 37] that could result in women discontinuing treatment early and preferring traditional medicine or spiritual options alone [24, 27, 35, 36, 39]. Similarly, the demographic context is necessary to anticipate the fact that breast cancer in SSA affects women at an early age [26, 30, 34–36, 39]. Finally, the economic context allows the financial capabilities of beneficiaries to be taken into account while serving as the basis for a relevant progressive recovery policy [27, 33–37, 39, 40, 43].

## Discussion

The aim of this study was to propose adapted framework for the global and systemic analysis of breast cancer control policies in SSA. The literature review and the policy Delphi allowed us to comprehensively identify the relevant items for an effective breast cancer policy in this region. These items can be grouped into 4 components: (1) stakeholders; (2) policy content; (3) policy process; and (4) context. An effective breast cancer policy content should focus on strengthening the different building blocks of a health system to address this disease. Likewise, adequate processes must be established to ensure that each piece of content is implemented effectively. We discuss some of the main points that emerged from our study.

First, our results highlight different stakeholders that should be considered in breast cancer policy in SSA. However, these stakeholders are numerous and should not be involved simultaneously in the policy. It is therefore essential to perform in-depth analysis of all stakeholders according to specific criteria (relevance, level of power, etc.) to determine the appropriate policy level at which they should be integrated. In particular, it is useful to underline the importance and impact that civil society organizations could have on public authorities for cancer control policy. Knowing that governments can change rapidly, these organizations are essential in advocacy and lobbying to keep cancer control on the political agenda. For example, they were very helpful in Senegal for the introduction of the law on free cervical and breast cancer care [48, 49].

Second, the governance and leadership building blocks are fundamental for an effective cancer policy. Our findings show that the governments of SSA countries have key responsibility for developing breast cancer policy. Similarly, they must ensure concerned stakeholders’ adherence and commitment while coordinating them because these stakeholders could be simultaneously autonomous and interdependent. Indeed, the SSA health system attracts a multitude of both national and international actors; however, when these actors are weakly coordinated and regulated by governments, the fragmentation of the health system increases. It is thus imperative that governments resume their central role in a transparent and accountable manner. In this respect, our results indicate how political will is essential to continuously mobilize the required resources and to promote the success of the policy. However, Alison T Mhazo and Charles C. Maponga invite us to go beyond the rhetoric of political will to better understand what motivates policy reforms in SSA. For them, political will is relevant, but other factors should also be considered, such as the distribution of costs and benefits of reforms, the form and expression of power among actors, the desire to win or stay in power, elite interests and political ideologies, and the diffusion of policies [50]. On the other side, the fragmentation of the system could be addressed by adopting an integrated approach for services, e.g. integration of breast cancer services into gynaecological services. This also helps to optimize resources and ensure effective pooling.

Third, regarding financing, our results point to the importance of calling for financing from different sources, especially external sources, to support this policy in a limited-resource context. However, a prior assessment of the real costs of implementing breast cancer programs is crucial for a sustainable growth in financial support, especially when the impact of funding (results, effects, etc.) can be demonstrated. In addition, multiple financing sources call for governments to develop pooling mechanisms to address the often divergent interests of funding partners [51]. These mechanisms are crucial to avoid fragmentation of the system and duplication of efforts and to ensure relevance and better coordination of interventions. In this respect, the principles of aid effectiveness [52] must be respected by the partners to achieve a coherent policy. This implies, among other things, harmonization between donors and their alignment with recipient country systems [51, 52]. This should be based on well-structured and sustainable public‒private partnerships that share health expenditures among all partners while emphasizing the role of each partner with accountability mechanisms [53]. Although private funding may be relevant for policy success, it is becoming increasingly scarce [54], and it is crucial for the public sector in SSA to increase its proportion of funding in accordance with the 2001 Abuja Declaration. This declaration stipulated that at least 15% of the annual budget of the states should be allocated to health [55]. In addition, our findings suggest that laws for free gynaecological cancer care, government subsidies, or universal health coverage are needed as a cost recovery mechanism. This is important to reduce out-of-pocket payments and further stresses the need for public financing in each country. For example, the Senegalese government implemented free chemotherapy in 2019 for all women with cervical and breast cancer, and three years later extended this to all anticancer treatments [48, 49]. This example demonstrates that access to cancer care for all is possible in a resource-limited country.

Fourth, with regard to human resources, our results highlighted the importance of a health workforce that should be available and accessible. It should be noted in this regard that we found no international agreement on the ideal ratio of health care professionals or services to the population for successful health programs, including cancer programmes. However, the WHO reported that countries with fewer than 23 doctors, nurses, and midwives per 10,000 population may fail to achieve coverage for some primary health care interventions [17]. To effectively control breast cancer in Sub-Saharan Africa, it is crucial to identify and agree on a professional, service, or technology-to-population ratio that each country can align with. Concerning health professional training, it is time for all countries in SSA to develop adequate training programmes to address the growing burden of cancer. According to the Global Cancer Observatory, by 2040, Africa will see its cancer incidence increase by 89% compared to 2020 [56]. Policy-makers must therefore be aware of the epidemiological transition and realign their health systems to address chronic diseases.

Fifth, the findings indicate that accessible, continuous, integrated, multidisciplinary, comprehensive, community-based, evidence-based and cost-effective cancer services are imperative. These services should provide breast cancer promotion, prevention, diagnosis, curative treatment and palliative care. However, there are some challenges due to the limited resources available in SSA. Which service should be implemented first? Is it appropriate to prioritize one service over another? In this regard, several authors of the papers included in our review suggested adopting a progressive implementation approach by starting with “treatment including palliation” services because more than 70% of women are diagnosed at a late stage of the disease [22, 23, 27, 30, 37, 36]. Treatment and palliative services aim to provide those diagnosed with the treatment they need today. Nevertheless, for the Delphi participants, SSA countries should prioritize “promotion and primary prevention” to limit advanced stages of cancer that require more resources. Indeed, these services are relevant for reducing advanced stages and future burden but do not address most breast cancers that do not have a dominant risk factor. The ideal is to develop systems that do both simultaneously. Accordingly, the WHO Global Breast Cancer Initiative recently called on countries to reflect on how to address breast cancer using a functional and sustainable approach that covers all three pillars of management, namely, early detection, prompt diagnosis, and treatment without abandonment. It also proposed evidence-based key performance indicators covering the three pillars to evaluate the breast-health care system: the proportion of TNM breast cancer cases diagnosed at stages 0–IV; the timeliness of confirmatory diagnosis of invasive breast cancer in patients with suspicious breast complaints; and the proportion of breast cancer patients who complete the recommended therapy without abandonment [3]. Another aspect less developed in the results is survivorship. Indeed, this aspect is often neglected in resource-limited countries, but it is important for comprehensive care during and after treatment. The introduction of a survivorship care plan is imperative in order to track the patient’s progress and outcomes, while considering, among other things, mental health, physical changes, social and economic reintegration etc [57]. Concerning the policy process, the priority assigned to an integrated patient pathway by the Delphi participants is in line with the literature [23, 30, 35, 36]. Indeed, without a clear and integrated care pathway, it would be almost impossible to achieve the expected policy outcomes. However, several countries in SSA do not have cancer care pathways. The BHGI has proposed a patient pathway for breast cancer [23], and the BCGI has proposed it in line with the three pillars of management [3]. SSA countries could rely on these proposals to develop their own pathways adapted to their reality.

Sixth, our results indicate that medicine and technology must be adequate and complete to allow service delivery to function appropriately. The focus should also be on the maintenance of the equipment once it is purchased. In many countries in SSA, equipment breakdowns are a major contributor to delays in care and to loss to patient follow-up [58, 59]. Regarding the list of essential oncology drugs, it is also crucial for decision-makers to examine their compatibility with the SSA population. These populations are underrepresented in the clinical trials that lead to the selection of these drugs. This could result in the use of drugs with little data on efficacy or adverse effects in this key population [60]. It would be advisable for actions to be implemented to ensure the representativeness of this population in future clinical trials.

Seventh, our findings highlight that health system information is central to evidence-based interventions and monitoring. Surprisingly, medical records, which are essential for patient follow-up and a valuable source of data, were not addressed by the document we reviewed. However, Su-Ying Liang et al. and Elizabeth Ngo et al. suggest that these records provide information on the stage of the cancer and comorbidities and ensure continuity of care [61, 62]. Therefore, breast cancer policies in SSA must establish a standardized medical record to improve care for women and ensure reliable data.

Finally, regarding the policy context, the various aspects identified should be considered to anticipate the risks of intervention failure, enhance beneficiaries’ adherence and meet their real needs. As suggested by the Delphi participants, it is vital to address the context as a whole, covering all the social determinants of health. This is also important in terms of reducing social inequalities in health and ensuring that people are treated holistically and that the root causes of the problem are addressed.

### Methodological strengths and limitations

To our knowledge, this study is the first to propose a comprehensive and systemic framework for breast cancer policy analysis in SSA. The two frameworks that served as a basis for analysing the information collected and designing our framework were internationally validated. The literature review combined with the Delphi survey reinforces the evidence of the identified items. Similarly, Delphi participants have significant experience with breast cancer in SSA. Regarding expert selection, participating in the World Cancer Congress had a double benefit: first, it facilitated access and adhesion to the Delphi survey of key and relevant actors; second, it helped to reinforce the methodology and legitimacy of the items highlighted in the framework.

This study has some limitations. First, the interactions between the components of a health policy may be more complex than presented in the framework. For ease of presentation, we adopted a static version of the framework, but we acknowledge that this fails to show the different interactions as they would be in reality. Second, the proposed framework is a comprehensive and an ideal preliminary framework based on our current knowledge and data collected. We are fully aware that all the elements may not necessarily be found in one country. Indeed, this proposed framework is intended to be further refined and revised after being ground-tested for breast cancer policy analysis. For this purpose, an in-depth analysis of the content of breast cancer policies currently adopted in SSA countries is now ongoing and case studies are planned to test the framework with the reality of at least two specific countries. Framework items could therefore be prioritized after these two phases, according to country needs. Third, the distinction between the two components of “policy content” and “process” may not be universally agreed upon. However, we proposed this distinction for analytical purposes, and the most important aspect for a breast cancer policy in SSA is to have all of the items identified, whether they are classified under content or process. Fourth, we were not able to obtain a representative sample of East African participants in the Delphi process, despite inviting several of them. However, we were pleased to have 3 of our participants with experience in East Africa (Uganda, Ethiopia, Kenya). Similarly, the literature review that helped initiate the framework covered the whole of sub-Saharan Africa.

## Conclusion

To ensure the success of breast cancer policy in SSA, it is crucial to adopt a holistic and systemic approach that addresses all identified interdependent components of the framework proposed in this study. This includes public policy that is adapted to the dynamic context and involves all relevant stakeholders. Walt and Gilson emphasized the need for political actors to consider these components when planning and analysing health policy in developing countries.

The analytical framework can be used for various purposes and can be adapted as new key elements are identified. This framework can assist stakeholders in planning, implementing, and evaluating cancer policies or programmes in resource-limited settings, particularly in SSA. Checklists can be created from the framework to aid in this process, and administrators can refer to it to ensure appropriate cancer services for patients at their facilities.

### Electronic supplementary material

Below is the link to the electronic supplementary material.

Supplementary Material 1

## Data Availability

Most of the data generated or analysed in this study are included in this document and its supplementary data. Only the questionnaire and the Delphi survey database are available upon request.

## Abbreviations

SSA: Sub-Saharan Africa
WHO: World Health Organization
GBCI: Global Breast Cancer Initiative
IARC: International Agency for Research on Cancer
BHGI: Breast Health Global Initiative
AOREC: African Organization for Research and Education on Cancer
SDGs: Sustainable Development Goals
QUATRO: Quality Assurance Team for Radiation Oncology
JBI: Joanna Briggs Institute
SANRA: Scale for the Assessment of Narrative Review Articles
WCG: World Cancer Congress
TNM: Tumour, Node, Metastasis
