# Supplementary material for: An analytical framework for breast cancer public policies in Sub-Saharan Africa: results from a comprehensive literature review and an adapted policy Delphi
Source: BMC Public Health. 2024 Jun 7;24:1535. doi: 10.1186/s12889-024-18937-5 (PMC11157826; doi:10.1186/s12889-024-18937-5)
Supplement: Supplementary file 1 — Supplementary Material 1 [file 12889_2024_18937_MOESM1_ESM.docx]

| ***Databases*** | ***Search equations*** |
| --- | --- |
| *PubMed (Filtres : 2015-2022 ; revue et revue systématique)* | *Breast cancer and (policy or guideline or strategy) and (sub-Saharan Africa or low-and middle-income countries)* |
|  | *(Breast cancer or breast tumor or breast carcinoma) and Management and (strategy or policy) and ((sub-Saharan Africa) or (Low-and middle-income countries))* |
|  | *Breast cancer and (low-middle income countries or sub-Saharan Africa) and (management or treatment option)* |
|  | *(Breast cancer[Title]) AND (service delivery[All Fields]) AND ((low-and middle income countries[All Fields]) OR (sub-Saharan Africa[All Fields)) AND (healthcare policy) Filters: Review, Systematic Review* |
|  | *(((Breast cancer[Title]) OR (Breast neoplasm[Title]) OR (Breast tumor[Title]) OR (Breast carcinoma[Title])) AND ((service delivery[All Fields]) OR (health workforce[All Fields]) OR (health information system[All Fields]) OR (technologies[All Fields]) OR (Medicines[All Fields]) OR (financing[All Fields]) OR (leadership[All Fields]) OR (governance[All Fields])) AND ((low-and middle income countries[All Fields]) OR (Subsaharan Africa[All Fields]))) Filters: Review, Systematic Review, from 2015 - 2022* |
|  | *(Health system) AND (Breast cancer) AND (effective management) AND (Subsaharan Africa)* |
|  | *(Health system) AND (Breast cancer policy) AND (Subsaharan Africa)* |
| *Cochrane review library (Filtres: 2015-2022)* | *'"Breast Cancer" AND "Health care policy" AND "Low Middle Income Country"'* |
|  | *"Breast Cancer" AND "Health care policy" OR "Policy maker " AND "Low Middle Income Country"* |
|  | *Breast Cancer in Record Title AND Health care policy in Title Abstract Keyword OR Policy maker in Title Abstract Keyword OR interventions in Title Abstract Keyword AND low and middle Income country in Abstract -* |
| *Scopus (Filtres: 2015-2022)* | *TITLE-ABS-KEY (Breast AND cancer AND health AND care AND policy AND low AND middle AND income AND country ) AND ( LIMIT-TO ( PUBYEAR , 2022 ) OR LIMIT-TO ( PUBYEAR , 2021 ) OR LIMIT-TO ( PUBYEAR , 2020 ) OR LIMIT-TO ( PUBYEAR , 2019 ) OR LIMIT-TO ( PUBYEAR , 2018 ) OR LIMIT-TO ( PUBYEAR , 2017 ) OR LIMIT-TO ( PUBYEAR , 2016 ) OR LIMIT-TO ( PUBYEAR , 2015 ) ) AND ( LIMIT-TO ( DOCTYPE , "re" ) )* |
|  | *Breast AND cancer AND sub-Saharan AND Africa AND management AND policy AND ( LIMIT-TO ( DOCTYPE , "re" ) ) AND ( LIMIT-TO ( PUBYEAR , 2022 ) OR LIMIT-TO ( PUBYEAR , 2021 ) OR LIMIT-TO ( PUBYEAR , 2020 ) OR LIMIT-TO ( PUBYEAR , 2019 ) OR LIMIT-TO ( PUBYEAR , 2018 ) OR LIMIT-TO ( PUBYEAR , 2017 ) OR LIMIT-TO ( PUBYEAR , 2016 ) OR LIMIT-TO ( PUBYEAR , 2015 ) )* |

**Additional file 1 :** Search equations

**Additional file 2**: Assessment of systematic reviews/meta-analysis by the Joanna Briggs Institute (JBI) critical appraisal checklist for systematic reviews

|  | ***Authors*** | | | | | | |
| --- | --- | --- | --- | --- | --- | --- | --- |
|  | *Elima Jedy-Agba et al.* | *Carolina Espina et al. 2017* | *Gbenonsi et al. 2021* | *Davies Adeloye et al. 2018* | *Cynthia Pomaa Akuoko et al. 2017* | *Tanimola Martins et al. 2020* | *Hand et al. 2021* |
| ***Items*** | *2016* | | | | | |  |
| ***Is the review question clearly and explicitly stated?*** | *Yes* | *Yes* | *Yes* | *Yes* | *Yes* | *Unclear* | *Yes* |
| ***Were the inclusion criteria appropriate for the review question?*** | *Yes* | *Yes* | *Yes* | *Yes* | *Yes* | *Yes* | *Yes* |
| ***Was the search strategy appropriate?*** | *Yes* | *Yes* | *Yes* | *Yes* | *Yes* | *Yes* | *Yes* |
| ***Were the sources and resources used for the study adequate?*** | *Yes* | *Yes* | *Yes* | *Yes* | *Yes* | *Yes* | *Yes* |
| ***Were the criteria for appraising studies appropriate?*** | *Yes* | *Yes* | *Yes* | *Yes* | *Yes* | *Yes* | *No appropriate* |
| ***Was critical appraisal conducted by two or more reviewers independently?*** | *Yes* | *Yes* | *Unclear* | *Unclear* | *Unclear* | *Yes* | *No appropriate* |
| ***Were there methods to minimize errors in data extraction?*** | *Yes* | *Yes* | *Yes* | *unclear* | *Yes* | *Yes* | *Yes* |
| ***Were the methods used to combine studies appropriate?*** | *Yes* | *Yes* | *Yes* | *Yes* | *Yes* | *Yes* | *Yes* |
| ***Was the likelihood of publication bias assessed?*** | *yes* | *Yes* | *Yes* | *Yes* | *Yes* | *Yes* | *No appropriate* |
| ***Were recommendations for policy and/or practice supported by the reported data?*** | *Yes* | *Yes* | *Yes* | *Yes* | *Yes* | *Yes* | *Yes* |
| ***Were the specific directives for new research appropriate?*** | *Unclear* | *Unclear* | *Yes* | *Yes* | *No* | *Yes* | *Unclear* |
| ***Overall appraisal*** | ***Include*** | ***Include*** | ***Include*** | ***Include*** | ***Include*** | ***Inlude*** | ***Inlude*** |

**Additional file 3 :** Assessment of narrative reviews by the Scale for the quality assessment of narrative review articles (SANRA)

|  | ***Items*** | | | | | | | ***Score*** | | ***Overall quality assessement*** | |
| --- | --- | --- | --- | --- | --- | --- | --- | --- | --- | --- | --- |
|  | ***Justification of the article’s importance for the readership*** | ***Statement of concrete aims or formulation of questions*** | ***Description of the literature search*** | ***Referencing*** | ***Scientific reasoning*** | ***Appropriate presentation of data*** | |  |  |  |  |
| ***Auteurs*** |  |  |  |  |  |  |  | | |  | |
| ***C. H. Yip et al. 2015*** | ***2*** | ***1*** | ***0*** | ***1*** | ***2*** | ***2*** | ***8*** | | | ***Medium*** | |
| ***O. Ginsburg et al. 2018*** | ***2*** | ***2*** | ***0*** | ***2*** | ***2*** | ***2*** | ***10*** | | | ***High*** | |
| ***Eva Johanna Kantelhardt, Herbert Cubasch, Claudia Hanson, 2015*** | ***2*** | ***2*** | ***0*** | ***2*** | ***2*** | ***2*** | ***10*** | | | ***High*** | |
| ***Kiven Erique Lukong et al.2017*** | ***2*** | ***2*** | ***0*** | ***2*** | ***2*** | ***2*** | ***10*** | | | ***High*** | |
| ***Flavia Zita Francies et al. 2020*** | ***1*** | ***1*** | ***0*** | ***2*** | ***2*** | ***2*** | ***8*** | | | ***Medium*** | |
| ***Lydia E. Pace et Lawrence N. Shulman, 2016*** | ***1*** | ***2*** | ***0*** | ***1*** | ***2*** | ***2*** | ***8*** | | | ***Medium*** | |
| ***Eleanor Black & Robyn Richmond, 2019*** | ***2*** | ***2*** | ***0*** | ***1*** | ***2*** | ***2*** | | | ***9*** | | ***High*** |
| ***Mutebi et al. 2020*** | ***2*** | ***2*** | ***0*** | ***2*** | ***2*** | ***2*** | | | ***10*** | | ***High*** |
| ***Martei et al., 2018*** | ***2*** | ***1*** | ***0*** | ***1*** | ***2*** | ***2*** | | | ***8*** | | ***Medium*** |

**Additional file 4 : List of Delphi participants who agreed to be disclosed**

| **Surname and first name** | **Current function** | **Headquarters country** |
| --- | --- | --- |
| **DILLE Issimouha** | **Cancer Programme Officer for WHO/AFRO** | **Burkina Faso** |
| **TRAORE Bangaly** | **Professor, Lecturer at Gamal Abdel Nasser University in Conakry and coordinator of the national cancer control programme** | **Guinea** |
| **Kabisa Mwala** | **Consultant Surgical Oncologist, Co-Chair Breast Subcommittee National Cancer Control Technical Working Group, Cancer Diseases Hospital – University Teaching Hospitals, Ministry of Health** | **Zambia** |
| **Cremelda Parkinson Pratt** | **CEO/Founder of Thinking Pink Breast Cancer Foundation** | **Sierra Leone** |
| **Samuel Onyinyechukwu Azubuike** | **Senior Lecturer , Breast cancer researcher National Open University of Nigeria** | **Nigeria** |
| **Naana Akyaa Asante** | **Founder and CEO of Isabella HealthCare Services and Embrace Society** | **Ghana** |
| **Hervé AKA** | **Project coordinator for the NGO Hope Life,**  **General Secretary of the Ivorian Coalition of Cancer Organisations** | **Ivory Coast** |
| **Vanderpuye Verna** | **Clinical oncology consultant at national center for radiotherapy, Breast cancer researcher** | **Ghana** |
